# Effects of Ionizing Radiation on Halogen-Bonded Dipyridyl-Naphthalenediimide Cocrystals

**DOI:** 10.1021/acs.cgd.5c00441

**Published:** 2025-06-10

**Authors:** Samantha J. Kruse, Tori Z. Forbes, Leonard R. MacGillivray

**Affiliations:** a Department of Chemistry, 4083 University of Iowa Chemistry Building, Iowa City, Iowa 52242, United States; b Department de chimie, 7321 Université de Sherbrooke, Sherbrooke, QC J1K 2R1, Canada

## Abstract

Cocrystals are promising modular materials that can contain aromatics as coformers with the ability to fluoresce upon radiation exposure for use in scintillation and dosimetry. The materials must be able to endure significant exposures to ionizing radiation, and there is currently a minimal understanding of atomistic criteria to enhance the structural stabilities of organic materials for such applications. The current study examines four cocrystals with a common molecular component as a naphthalene backbone-dipyridyl-naphthalenediimide (**NDI**) that interact with the halogen-bond (XB) donors **I**_**2**_, diiodobenzene (**DIB**), and diiodotetrafluorobenzene (**DITFB**). Powder X-ray diffraction was used to assess changes in crystallinity upon gamma (γ) irradiation and was combined with density functional theory calculations that provide atomistic-level differences in bond lengths, packing, and electrostatic energy surfaces. The presence of the aromatic groups did not affect the structural integrity of cocrystals; rather, the combination of both stronger primary and secondary interactions involving the XB systems ((**NDI**)·(**X-donor**), where **X-donor** = **I**_**2**_, **DIB**, or **DITFB**) supported an increase in structural integrity. The results provide likely trends (involving factors such as aromaticity, secondary interactions, and packing) that impact the design of multicomponent scintillators and/or radiation shielding materials. Importantly, this study found that aromaticity is not necessary to increase structural stability; rather, the primary and secondary interactions that hold the organic molecules together are of importance.

## Graphical abstract

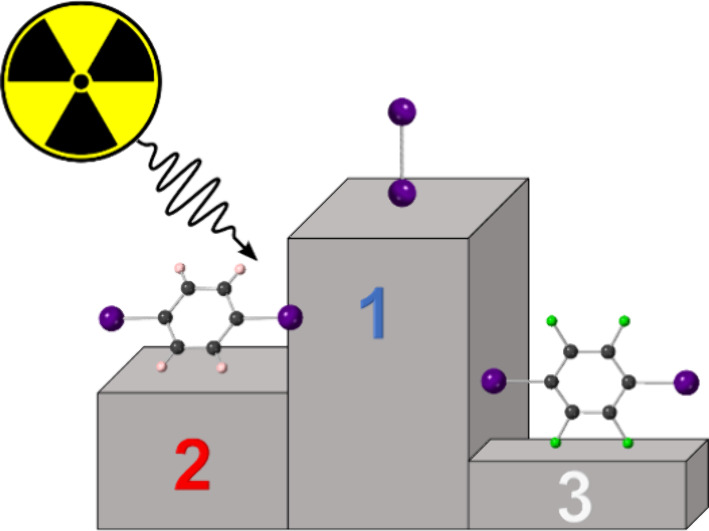

## Introduction

Organic materials are widely used in applications involving ionizing radiation fields. For example, at the International Space Station, organic coatings such as polyethylene are employed for radiation shielding owing to affordability and ease of handling.^1^ Within the field of dosimetry and radiation detection, three of the most common solid-state organic scintillators include anthracene,^2^ naphthalene,^3,4^ and *trans*-stilbene,^5^ which leverage a material's fluorescent properties. While these compounds are generally resistant to damage caused by ionizing radiation, the stabilities are lower than what is typically observed for inorganic materials owing to higher cross sections (i.e., increased probability of interaction with radiation) and densities.^6^ Improving the structural stability of solid-state organic materials upon exposure to ionizing radiation is critical for future advancements in dosimetry and radiation shielding.

Radiation effects on polymeric and other single-component crystalline materials have been explored to provide insights into the mechanisms of degradation and damage. For polymers, ionizing radiation results in bond scission and cross-linking.^7–9^ The change in the chemical environment leads to a change in material properties upon exposure to ionizing radiation (i.e., tensile strength, fluorescence, and decrease in crystallinity).^10,11^ For polyethylene, it is the hydrogen-rich nature of the material that is thought to prevent nuclear fragmentation and to limit harmful radiation effects. As for single-component scintillators, the conjugated nature of the material supports radiation resistance, given electron delocalization that prevents bond breakage.^9^ At the molecular level, there is a good understanding of how organic materials that resist radiation damage might be achieved; however, additional features, such as intermolecular interactions and structural packing, may influence structural stability. Therefore, additional efforts are needed to explore these features for the rational design of structurally sound organic materials.

Cocrystals are a class of binary organic materials that have high tunability and may offer enhanced radiation resistance. Work of Quaranta et al. reported that binary polymeric materials provide enhanced structural stability for materials in ionizing radiation fields.^7^ The crystalline nature of cocrystals means that the materials can be structurally characterized and therefore provide precise insight into bond types, bond lengths, and bond strengths, as well as packing arrangements in relation to radiation structural stability. These solid-state materials can be rationally designed for a multitude of applications including optoelectronics,^12,13^ pharmaceuticals,^14^ solid-state reactions,^15^ and the construction of more recently “radiation-resistant” materials.^10,11^ However, to rationally design materials with the intention of radiation resistance, changes upon irradiation at an atomistic level for a range of chemical components must be investigated first.

The current study explores changes in the crystalline structure owing to exposure to ionizing (γ) radiation within a class of halogen-bonded (XB) binary cocrystals. The binary cocrystals include naphthalenediimide (**NDI**) as the XB acceptor that is paired with the XB donors **I**_**2**_, diiodobenzene (**DIB**), and diiodotetrafluorobenzene (**DITFB**). The solids constitute binary cocrystals (**NDI**)·(**X-donor**) (where XB donor = **I**_**2**_, **DIB**, or **DITFB**) (Scheme 1). The materials were chosen owing to the highly conjugated naphthalene backbone of **NDI** and its capabilities as an XB acceptor, which provides a means to systematically evaluate changes in the stability of each cocrystal. Coformers were chosen based on the study by Mazzeo et al.^16^ wherein the XB donors provide means to evaluate aromaticity,^17,18^ σ-hole potentials,^19^ and varying secondary interactions.^20^ Upon syntheses and characterization, the cocrystals were exposed to γ-radiation (10 kGy; ∼200 years of cosmic radiation exposure) and then evaluated postirradiation to determine the structural stability of each cocrystal. Electrostatic mapping and modeling were used to further delineate structural changes at the atomistic level.

**Scheme 1:**
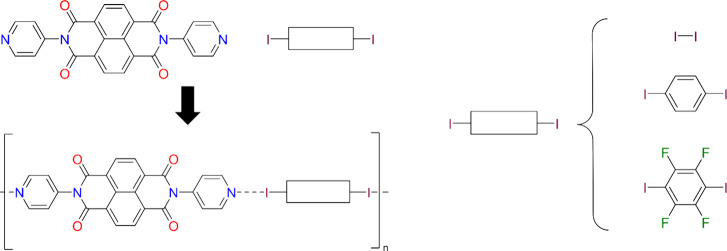
Wire Representation of Reactant **NDI** and XB Donor Forming Cocrystals^a^

a The XB donor represented, as described at the right, is either **I**_**2**_, diiodobenzene (**DIB**), or diiodotetrafluorobenzene (**DITFB**).

## Experimental Section

### Sample Preparation

Coformers were purchased from Sigma-Aldrich and recrystallized to ensure that the samples used in this study contained no impurities. **NDI** was synthesized as described previously by Mazzeo et al.^16^ (^1^H NMR, Figure S1). The reactant (**NDI**) and the coformer (**I_2_**, **DIB**, or **DITFB**) were combined in a 1:1 (reactant:coformer) ratio and ground together via liquid-assisted grinding (LAG) using 50 μL of DMF for 60 min at 15 rpm with two ball bearings using a Mixer Mill MM Vario 500. Once completed, samples were transferred to 20 mL scintillation vials, dissolving the ground material in 4 mL of DMF, heated for 1 h at 100 °C, and stirred to ensure full dissolution. Once removed from the heat, they were then capped to allow for slow evaporation to take place. Recrystallization measures were taken to ensure a consistent microcrystalline product, whereas both micro- and nanocrystalline products are present after LAG. Once crystallized and evaporated to complete dryness, samples were ground into a fine polycrystalline powder for approximately 5 min each using an agate mortar and pestle. Sample conversion was then characterized using PXRD and matched via calculating the powder pattern of each material using the .cif file and comparing it to the powder pattern collected experimentally. CSD refcodes for (**NDI**)·(**I**_**2**_), (**NDI**)·(**DIB**), and (**NDI**)·(**DITFB**) include YAHYIT, YAHYUF, and YAHYOZ, respectively.

### Powder X-ray Diffraction

All cocrystals were mixed with NaCl as an internal standard to confirm instrument and sample alignment and to assist with semiquantitative analysis of the material. NaCl was chosen because the diffraction peaks did not interfere with any of the powder pattern features of the samples. Each sample contained 12 mg of material that was ground with 3 mg of NaCl for 5 min to form a fine powder and then sieved to create a homogeneous mixture. These samples were analyzed on a Bruker D-5000 powder X-ray diffractometer (Cu Kα = 1.54 Å) equipped with a LynxEye solid-state detector to determine the purity of the sample. Scans were performed from 5 to 60 °2θ with a step size of 0.02 °2θ and a count time of 0.5 s/step. Experimental patterns were compared before and after γ-radiation exposure.

### Differential Scanning Calorimetry

A DSC Q100 (TA Instrument, USA) calorimeter was heated from 50 to 400 °C at 5 °C·min^–1^ to assess phase transitions of each cocrystal. Calibration was carried out with an indium and sapphire standard, and an empty, hermetically sealed aluminum pan was used as a reference. Approximately 5 mg of each cocrystal was weighed using a Toledo microbalance with 1 μg accuracy. The sample was placed in an aluminum pan, capped with an aluminum lid, and hermetically sealed. Data was analyzed using the free TRIOS version 5.1.1 software by TA Instruments. All phase transitions match previously reported transitions by Mazzeo et al.^16^ (Figures S5–S7).

### γ-Radiation

CAUTION: ^137^Cs is a radioactive γ-emitter. Radiation experiments were carried out by trained personnel in a licensed research facility.

All powders (0.05 g) were added to individual 0.5 dram borosilicate glass vials. The vials were completely evacuated, backfilled with Argon gas, and then tightly sealed to prevent the formation of reactive O_2_. Samples were irradiated by a ^137^Cs monoenergetic source (*E*_γ_ = 0.667 MeV) housed at the University of Iowa Free Radical and Radiation Facility. The total dose delivered to the samples was 10.00 kGy (8.45 h). Samples were safe to handle immediately following irradiation. The materials were further analyzed using PXRD after irradiation using the same methodology as described above.

### Electrostatic Potential Mapping

Electrostatic potential energy surfaces were determined using a Spartan’24 molecular modeling program.^21^ Single-crystal files were uploaded to Spartan’24 to provide the structural details for the calculation. Imported .cif files were then geometrically optimized in Spartan’24 and then DFT calculations using ground-state DFT calculations at the B3LYP/6-311++G** level. CSD refcodes for (**NDI**)·(**I**_**2**_), (**NDI**)·(**DIB**), and (**NDI**)·(**DITFB**) include YAHYIT, YAHYUF, and YAHYOZ, respectively.

## Results and Discussion

### Structural Characteristics

All cocrystals reside in the same space group (triclinic, *P*-1) (Table 1). The components of each cocrystal are bound by C–I···N XB involving either **I**_**2**_, **DIB**, or **DITFB** and the pyridyl ring of **NDI**. Each cocrystal shows that the pyridyl ring twisted approximately 90° to the naphthalene backbone, which means that there is generally a lack of conjugation between the backbone and pyridyl ring. All cocrystals form infinite chains sustained by the C–I···N XB along the *c* axis with offset π–π stacking interactions between the faces of adjacent **NDI** groups along the *a* axis. For (**NDI**)·(**DITFB**), minimal offset π–π stacking exists between adjacent **NDI** and **DITFB** molecules in addition to π–π stacking between neighboring **NDI** molecules along the *a* axis. The angles between the I and the pyridyl ring of **NDI** are 169.5, 173.8, and 178.6°, respectively, for (**NDI**)·(**DIB**), (**NDI**)·(**I**_**2**_), and (**NDI**)·(**DITFB**) (Figure 1).

**Table 1:** Crystallographic Unit Cell Data for Each Cocrystal

	**(NDI) (**I_2_**)**	**(NDI) (DIB)**	**(NDI) (DITFB)**
space group	*P*-1	*P*-1	*P*-1
*a* (Å)	9.2012(12)	5.3756(8)	9.8430(14)
*b* (Å)	10.2240(13)	10.9675(16)	1.7528(15)
*c* (Å)	11.8318(15)	11.5660(16)	13.5633(19)
α (°)	78.084(2)	74.520(2)	83.888(2)
β (°)	84.420(2)	78.023(2)	85.711(2)
γ (°)	84.457(2)	83.208(2)	78.791(2)
*V* (Å^3^)	1081.9(2)	641.39(16)	1398.8(3)
ρ_calc_(g/cm^3^)	2.069	1.942	1.952

**Figure 1:**
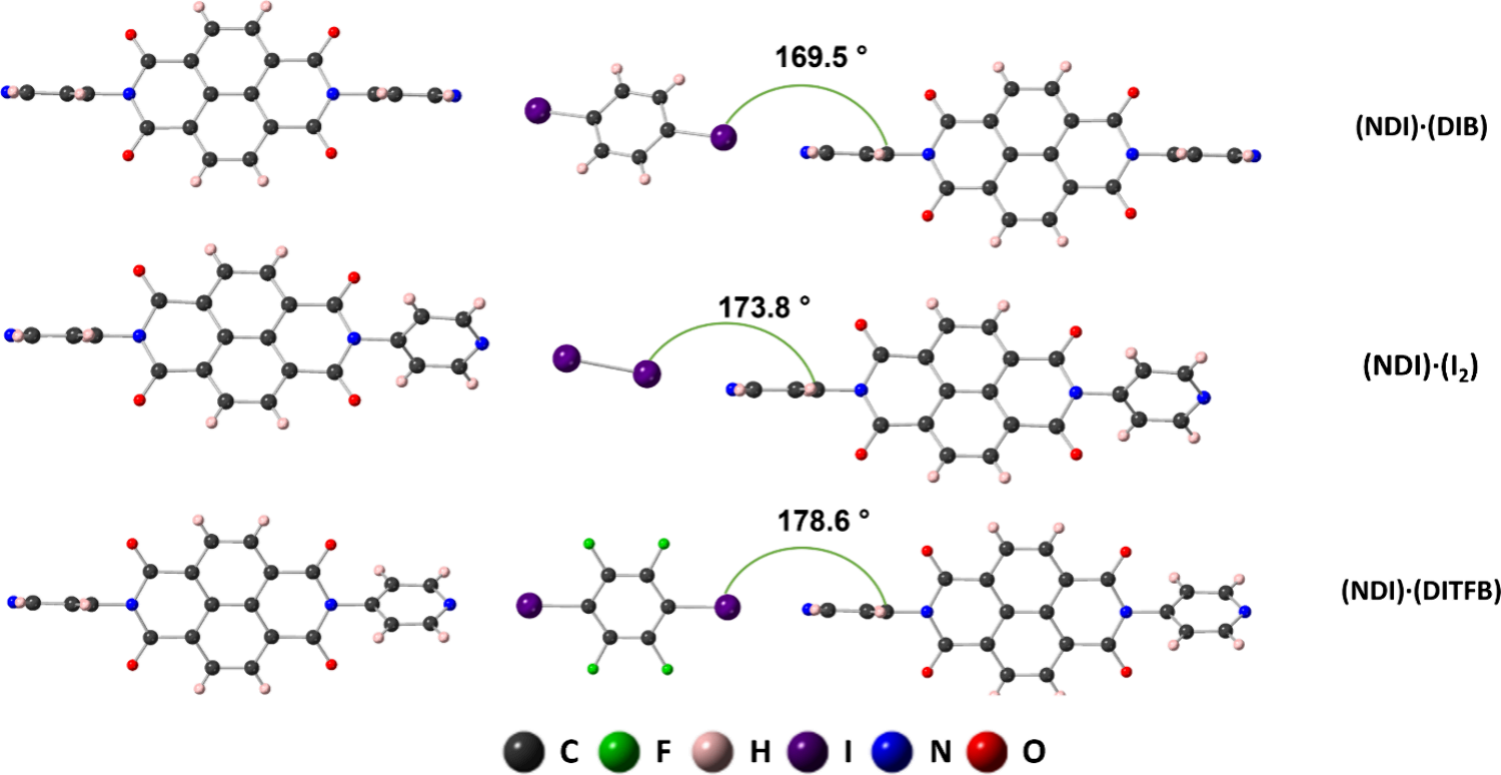
Ball-and-stick models with associated angles between the I and aromatic ring of the bonded **NDI**. Colors of elements are listed in the legend.

### Evaluation of Structural Stability

Changes in crystallinity were determined via semiquantitative PXRD examination wherein an internal standard of NaCl was used to assess changes in peak intensity (Figure 2 and Figures S9 and S10). In general, each cocrystal exhibited a decrease in the crystallinity upon γ-irradiation (Table 2). Interestingly, the cocrystal with components can be considered to exhibit the least aromaticity in the coformer, and (**NDI**)·(**I**_**2**_) displayed the smallest decrease in crystallinity (17.1%), followed by (**NDI**)·(**DIB**) (21.5%) and (**NDI**)·(**DITFB**) (48.3%). The following trend in structural stability is as follows:

$$I_{2}>\mathrm{DIB}>\mathrm{DITFB}$$

**Table 2:** Average Percent Decrease in Crystallinity upon γ-Irradiation for Each Cocrystal Calculated from Tables S1–S3

**cocrystal**	**percent decrease in crystallinity**
(**NDI**) (**I_2_**)	17.1
(**NDI**) (**DIB**)	21.5
(**NDI**) (**DITFB**)	48.3

**Figure 2:**
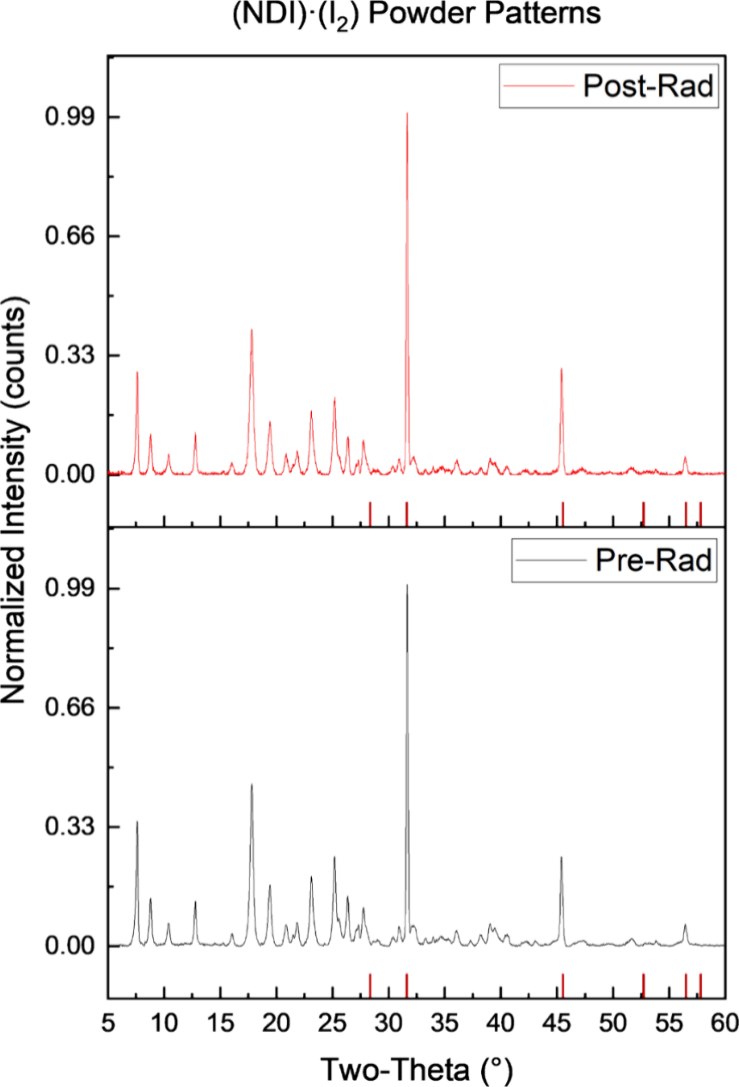
Normalized powder patterns pre- (black) and postirradiation (red) for (**NDI**)·(**I**_**2**_). NaCl standard powder pattern peaks are labeled with red tick marks.

An in-depth analysis of the bonding and packing of each cocrystal is discussed to understand the structural stability trend above.

With the solids being isostructural, a comparison of density is

$$I_{2}>\mathrm{DITFB}>\mathrm{DIB}$$

The densities, therefore, do not follow the trend of the structural stability. An additional analysis of the effects of radiation includes examination of *hkl* planes associated with the peak of the highest percent intensity change. Interestingly, the corresponding *hkl* plane for (**NDI**)·(**I**_**2**_) is the (221) plane, which dissects the layers of both **NDI** and **I**_**2**_. Marginal electron density cuts through the H of the peripheral pyridyl rings of **NDI** (Figure 3). In contrast, both (**NDI**)·(**DIB**) and (**NDI**)·(**DITFB**) exhibited *hkl* planes with the largest decrease in intensity that slices horizontally through **NDI** and the halogenated coformer (Figures S14 and S15, respectively).

**Figure 3:**
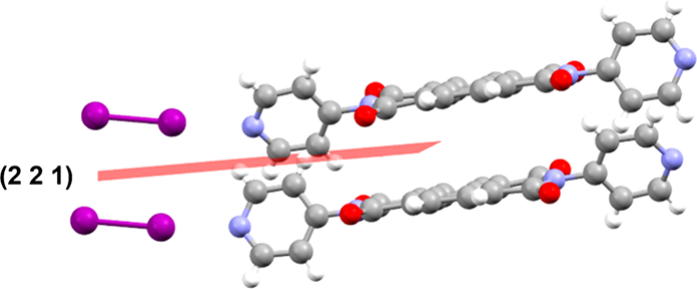
(221) plane of (**NDI**)·(**I**_**2**_) associated with the largest diffraction peak decrease upon irradiation. Hydrogen, carbon, nitrogen, oxygen, and iodine are depicted as white, gray, blue, red, and purple, respectively.

The loss in electron density in the regions for each cocrystal aids our understanding of a mechanism of decomposition due to irradiation. Specifically, it is known within organic materials with aromatic rings that H-abstraction and loss in aromaticity can occur upon irradiation.^22^ Moreover, the corresponding *hkl* planes suggest a region wherein there is loss of aromaticity in each cocrystal. For (**NDI**)·(**I**_**2**_), the (221) plane associated with the largest decrease in crystallinity is observed for the plane slicing through the H of the pyridyl ring between adjacent NDI molecules, which suggests a loss of the H bound to the pyridyl ring and a potential loss in aromaticity. For the cocrystals (**NDI**)·(**DIB**) and (**NDI**)·(**DITFB**), the *hkl* planes associated with the greatest decrease in crystallinity are associated with a slice through the central aromatic naphthalene unit of the NDI molecules, the pyridyl rings, and the aromatic XB donors. Consequently, the cocrystals are expected to involve general losses in electron density of the aromatic units, which suggests removal of an electron from the delocalized systems. For the latter two cocrystals with electron density cutting through the aromatic units in each respective *hkl* plane, it is probable that the aromatic unit no longer remains flat, leading to the puckering of the formerly planar units, as suggested by prior literature.^23,24^ Therefore, the puckering of the formerly planar units removes the aromaticity present previously in the system; thus, the electron delocalization cannot take place, ultimately decreasing the structural stability of the material.

To gain an atomistic perspective into structural effects, we compared differences between each cocrystal to understand the importance of aromaticity, differences in σ-hole potentials, and structural stability with increased conjugation. Thus, the application of electrostatic potential mapping was used to determine possible influences of differences in σ-holes across the series of cocrystals (Figure 4). As revealed by the blue color region, the cocrystal (**NDI**)·(**I**_**2**_) appears to exhibit the strongest σ-hole potential, followed by (**NDI**)·(**DITFB**) and (**NDI**)·(**DIB**). Comparing (**NDI**)·(**DITFB**) and (**NDI**)·(**DIB**), the presence of electron-withdrawing groups likely increases the σ-hole potential, although (**NDI**)·(**DITFB**) is considered the least structurally resistant to changes of irradiation. The XB interaction may, thus, be one of the important factors toward structural stability. The observation is consistent with previous work wherein primary interactions are not necessarily the determining factor for structural stability upon irradiation.^11,25^

**Figure 4:**
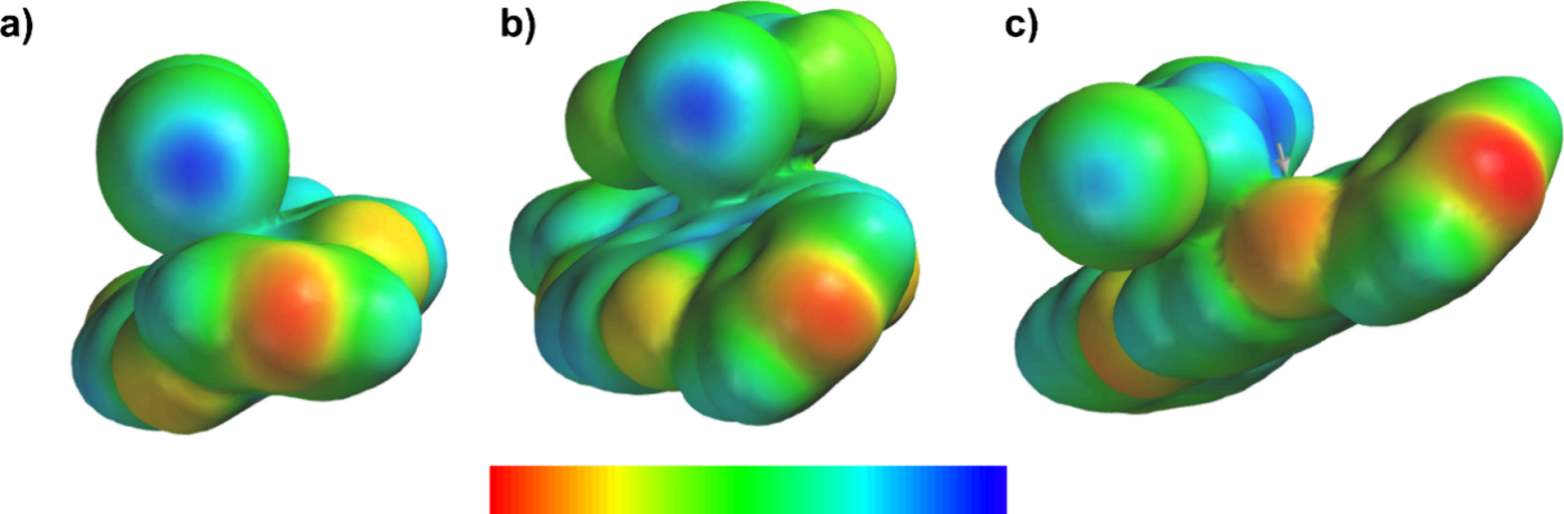
Electrostatic potential maps: (a) (**NDI**)·(**I**_**2**_) *V*_max_ = 186 kJ, (b) (**NDI**)·(**DITFB**) *V*_max_ = 180 kJ, and (c) (**NDI**)·(**DIB**) *V*_max_ = 147 kJ. Negative and positive electrostatic potentials are colored red and blue, respectively.

In terms of secondary interactions, (**NDI**)·(**I**_**2**_) displays C–H···O interactions between neighboring **NDI** molecules (2.445(2) Å) (Figure 5a). Face-to-face π–π stacking is largely absent, although it is present in the other cocrystals. Comparing the packing between (**NDI**)·(**DITFB**) and (**NDI**)·(**DIB**), the solid that contains **DIB** exhibits a more direct face-to-face overlap of the aromatic ring system with one of the pyridyl rings of **NDI** (2.733(2) Å) (Figure 5b). Additional interactions include the π–π stacking interaction between **NDI** molecules along the *a* axis (3.477–3.662(2) Å) involving the naphthyl groups. For (**NDI**)·(**DITFB**), the most prominent interaction between **NDI** and **DITFB** is of the C–F π–π stacking type (3.322 Å) (Figure 5c). Additional π–π stacking interactions in (**NDI**)·(**DITFB**) are associated with nearby **NDI** groups along the *a* axis (3.549–3.669 Å). However, the largest difference between the **NDI** groups in these two systems is that along the *a* axis, the **NDI** molecule flips to have pyridyl rings twisted to one another in (**NDI**)·(**DITFB**), preventing π–π stacking between these rings. As described in previous work, the presence of π–π stacking interactions provided increased structural stability of cocrystalline systems. Therefore, the absence of π–π stacking interactions between the pyridyl rings between layers of (**NDI**)·(**DITFB**) may be owed to its decreased structural integrity.^10,11^

**Figure 5:**
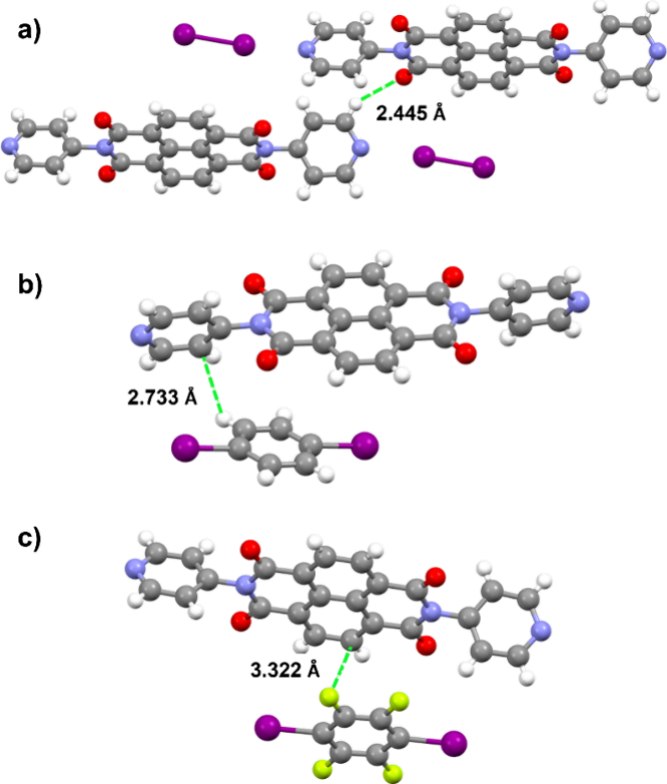
Distances of nearest neighbor interactions: (a) (**NDI**)·(**I**_**2**_), (b) (**NDI**)·(**DIB**), and (c) (**NDI**)·(**DITFB**).

Thus, strong XB interactions are important determinants for increased structural stability, comparatively for these cocrystals; however, the combination of strong primary and secondary interactions contributed to structural stability. Furthermore, from an examination of the *hkl* plane in relation to the largest decrease in peak intensity upon irradiation of (**NDI**)·(**I**_**2**_) (Figure 3), evaluating the interactions present in this system, as depicted in Figure 4a, if H-abstraction occurs in this system (a common phenomenon upon irradiation of aromatic organic molecules),^11,26,27^ then it is likely that the C–H···O interactions prevent the complete breakage of this C–H bond from **NDI**. Thus, with the loss of electron density confined to locations where aromaticity is not directly lost upon irradiation, the presence of secondary interactions can prevent free radical formation in the system, further preventing the breakdown of material of interest.^11^

## Conclusions

Herein, our study reports the effects of γ-radiation on a series of three XD cocrystals with naphthalenediimide backbones, including (**NDI**)·(**I**_**2**_), (**NDI**)·(**DIB**), and (**NDI**)·(**DITFB**), using PXRD and crystallographic information regarding the packing of each system. The systems provide insight regarding the importance of XB donors, aromaticity, packing arrangements, and secondary interactions that aid in establishing structural integrity upon exposure to ionizing radiation. It has been determined that aromaticity is not necessary to increase structural stability; rather, interactions that hold the organic molecules together are of importance. As such, this provides new information where prior studies explain the importance of aromaticity aiding in material structural integrity upon radiation exposure;^10,11^ however, these results further complicate what material characteristics can be used for rational design applications. The materials provide a potential new class of scintillating materials with a naphthalene backbone to be further explored, where rational design and material tunability can be achieved upon cocrystallization.

Though prolonged exposure leads to the breakdown of materials over time, providing material decomposition and long-range disorder, it remains necessary to evaluate the atomistic-level changes that lead to such changes. Additionally, the information provides insight into atomistic-level changes that can be integrated to rationally designed materials with the intent of stability upon radiation exposure. We believe that our study contributes to an understanding of atomistic properties such as changes in σ-hole potentials, aromaticity of XB donor coformers, and differences in secondary interactions to aid in structural stability. Future work aims to obtain probe decompositions of the materials to understand thresholds where the crystalline materials can be used for radiation detection and shielding.

## Supplementary Material



## Notes

Details of PXRD, ^1^H NMR spectroscopy, and DSC data (PDF)
