# Supplementary material for: Effects of Ionizing Radiation on Halogen-Bonded Dipyridyl-Naphthalenediimide Cocrystals
Source: Cryst Growth Des. 2025 Jun 10;25(13):4968–73. doi: 10.1021/acs.cgd.5c00441 (PMC12232308; doi:10.1021/acs.cgd.5c00441)
Supplement: Supplementary file 1 [file cg5c00441_si_001.pdf]

Effects of Ionizing Radiation on Halogen-Bonded Dipyridyl-Naphthalenediimide Cocrystals

Samantha J. Kruse[a], Tori Z. Forbes[a], Leonard R. MacGillivray[a,b]*

[a] Department of Chemistry, University of Iowa Chemistry Building, Iowa City, Iowa, 52242, USA

[b] Department de chimie, Université de Sherbrooke, Sherbrooke, QC, J1K 2R1, Canada

Table of Contents

Material characterization

^{1}H-NMR……....……………………………………………………………………………..3

Powder X-ray Diffraction…..……...……………………….……………….…………………4

DSC………………………………………………………………………………………...7

Post-Irradiation Analysis

Normalized pre- and post-irradiation diffractograms……………………...………..…………9

Pre- and post-irradiation diffractograms raw files………………………….........………....…12

Calculated percent decrease in crystallinity…………………...…………..…...……......….…13

***hkl* planes……………...…...……….……………………………………………………14**

^{1}H-NMR

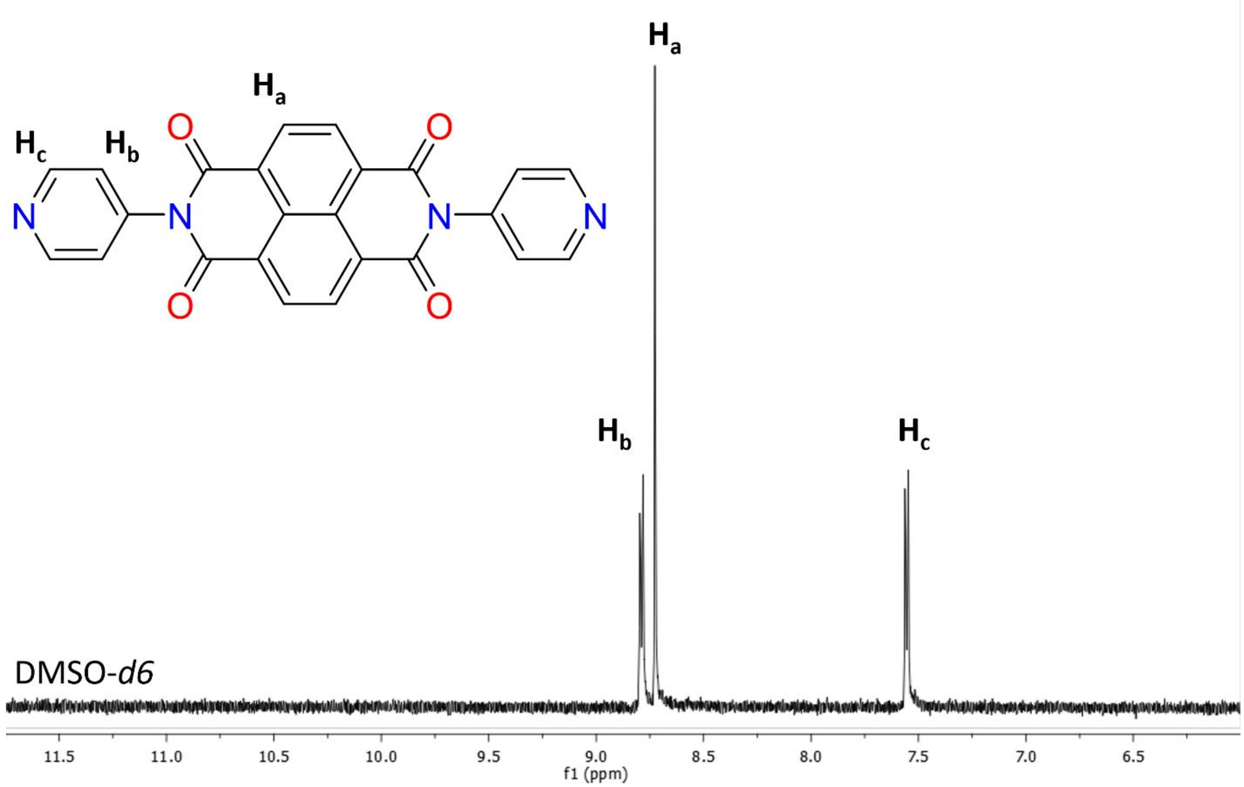

Figure S1. ^{1}H-NMR of naphthalenediimide (**NDI**) in DMSO-*d6*.

Powder X-ray Diffraction

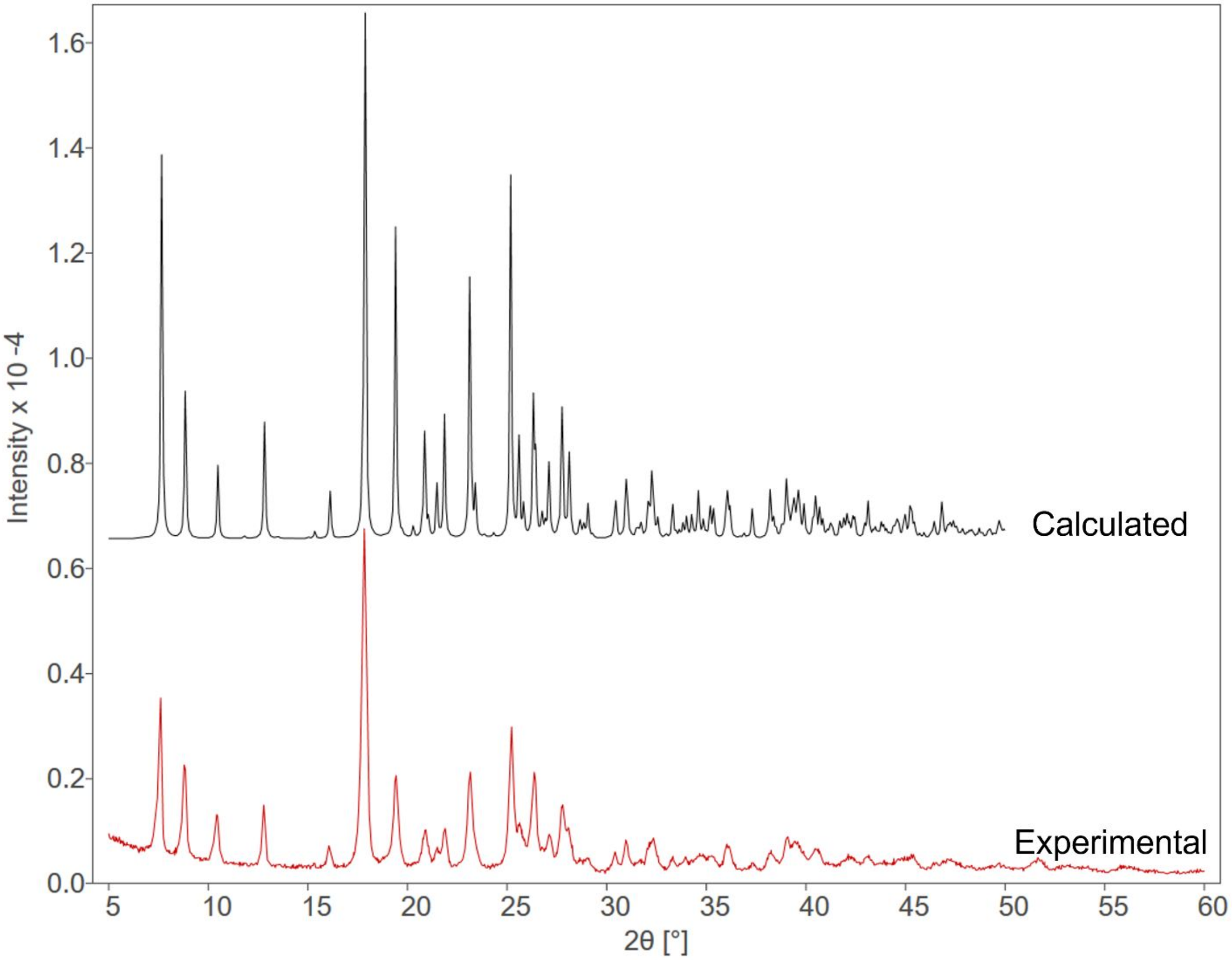

Figure S2. Phase matching of synthesized (**NDI**)·(**I_2**) (red) with calculated powder pattern (black, CSD refcode YAHYIT).

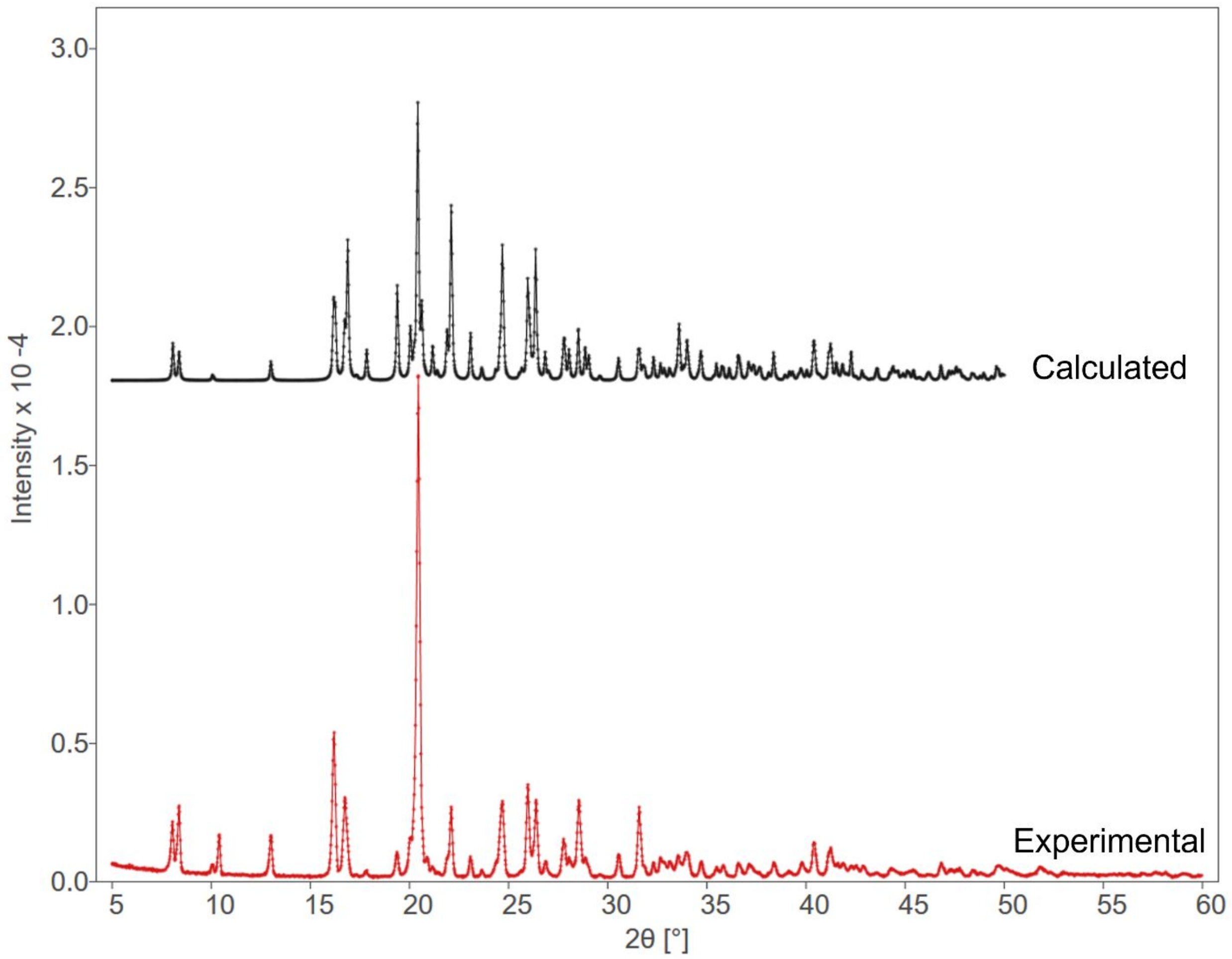

Figure S3. Phase matching of synthesized (**NDI**)·(**DIB**) (red) with calculated powder pattern (black, CSD refcode YAHYUF).

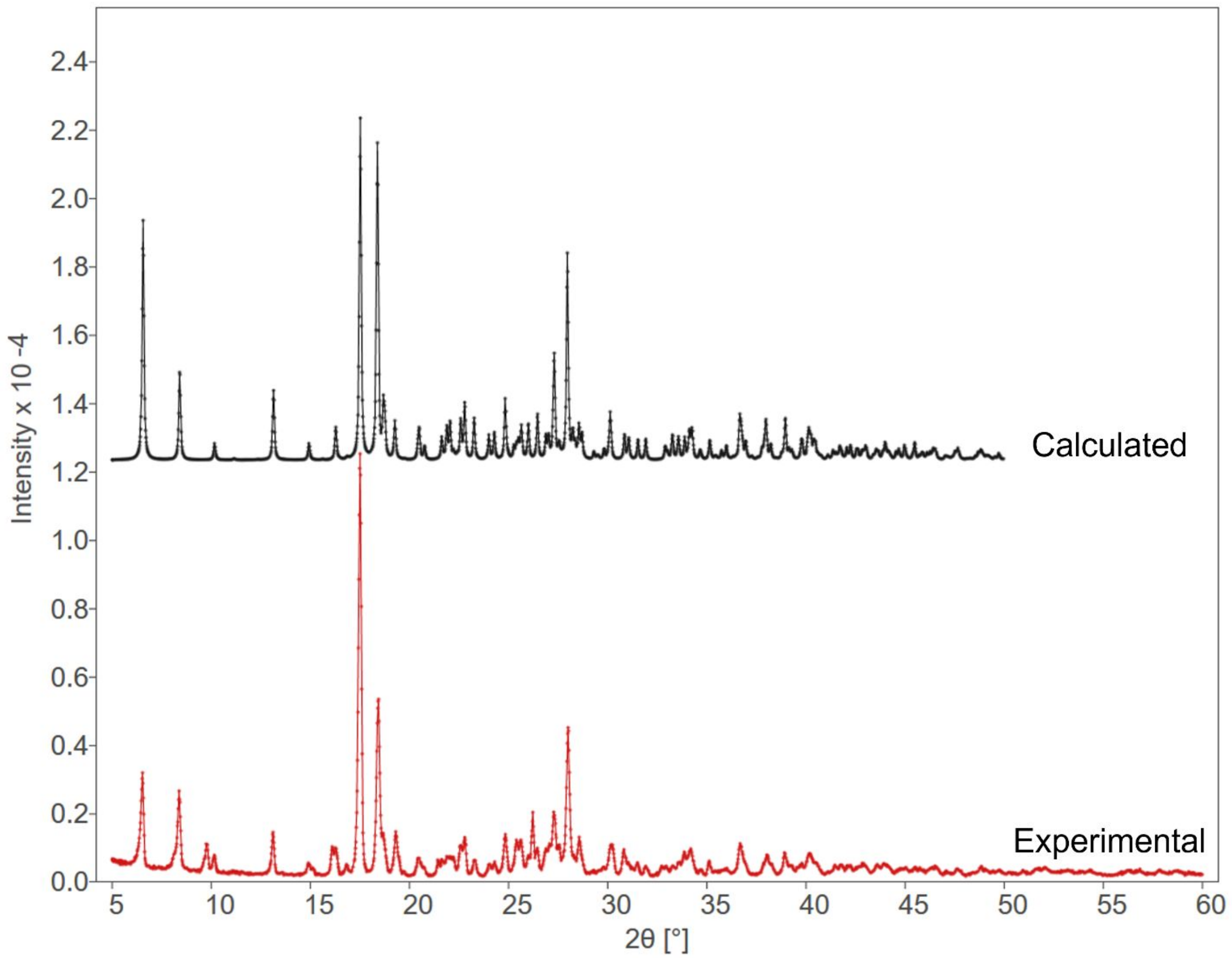

Figure S4. Phase matching of synthesized (**NDI**)·(**DITFB**) (red) with calculated powder pattern (black, CSD refcode YAHYOZ).

DSC

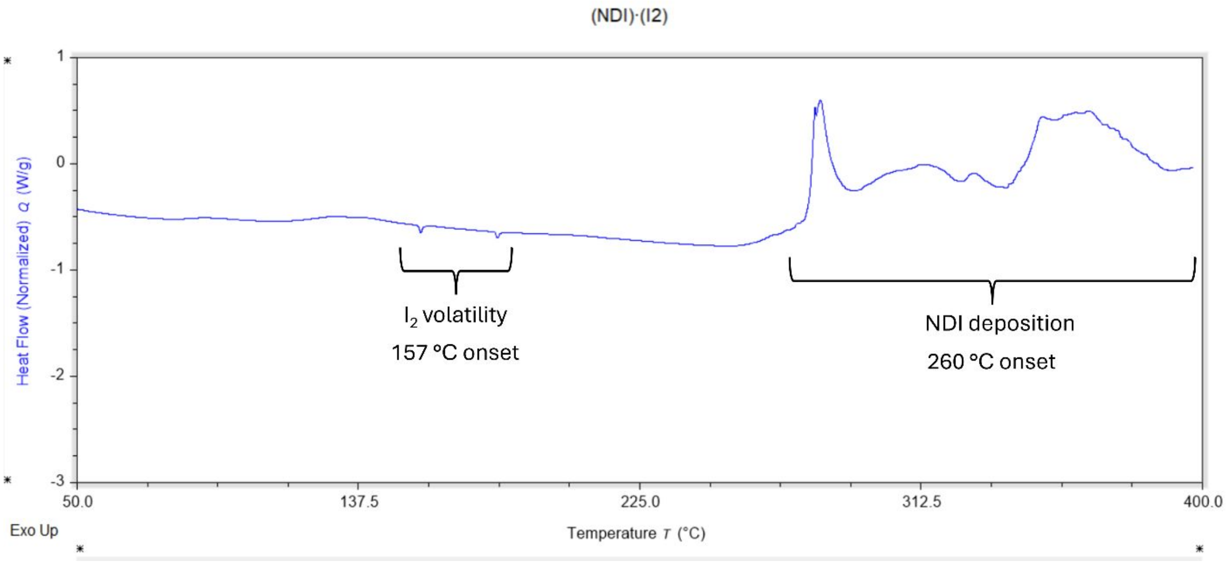

Figure S5. DSC thermogram of (NDI)·(I_2).

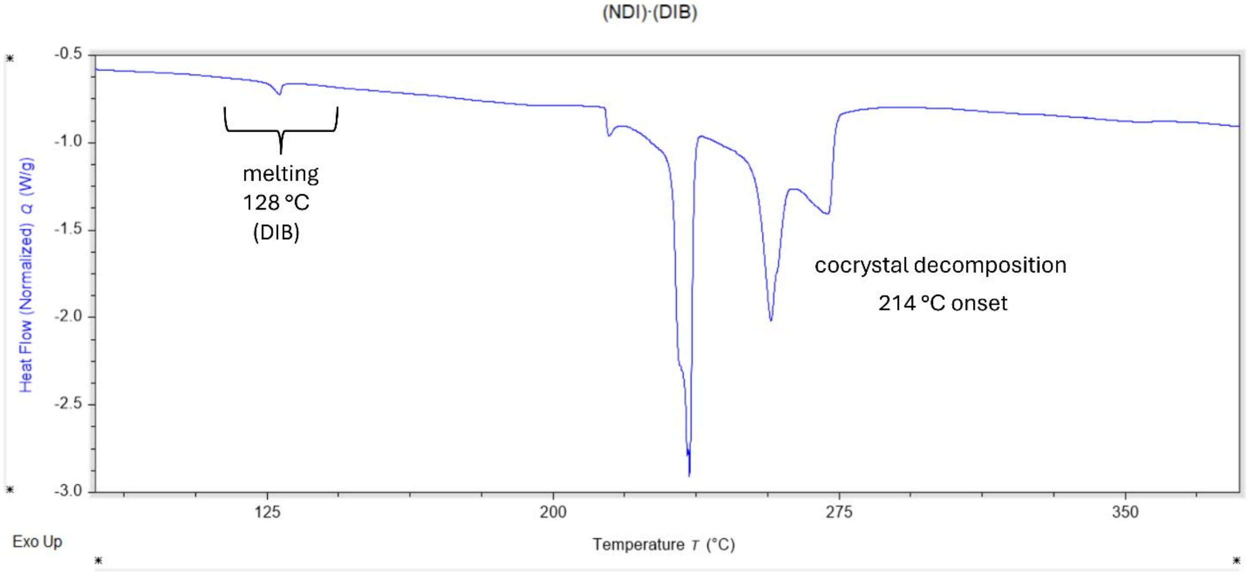

Figure S6. DSC thermogram of (NDI)·(DIB).

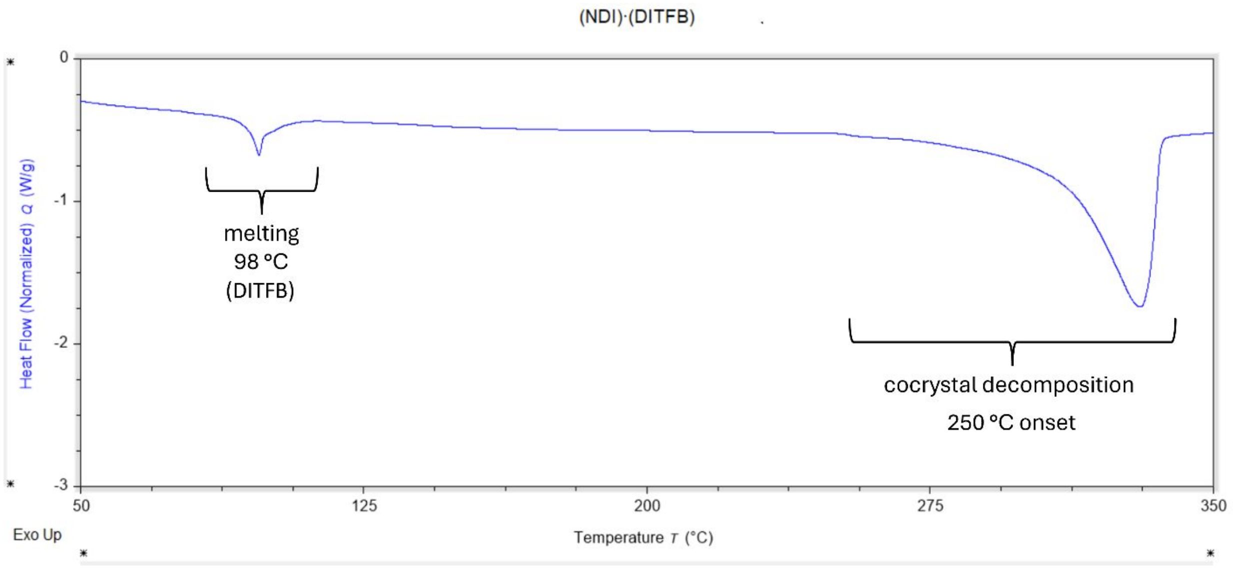

Figure S7. DSC thermogram of (NDI)·(DITFB).

Normalized pre- and post-irradiation diffractograms

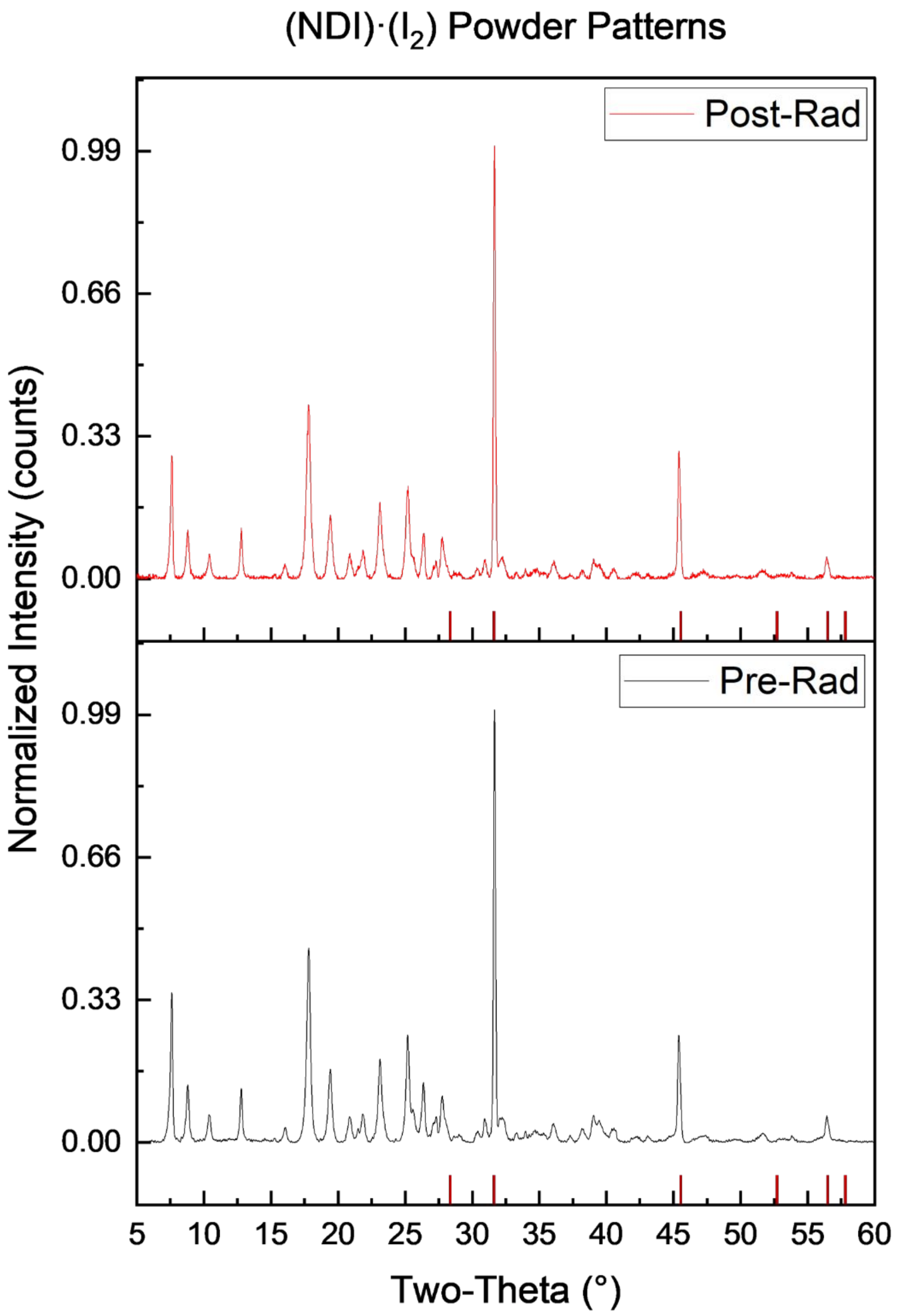

Figure S8. Normalized powder patterns pre- (black) and post-irradiation (red) for (**NDI**)·(**I$_2$**). NaCl standard powder pattern peaks are labeled with red tick marks.

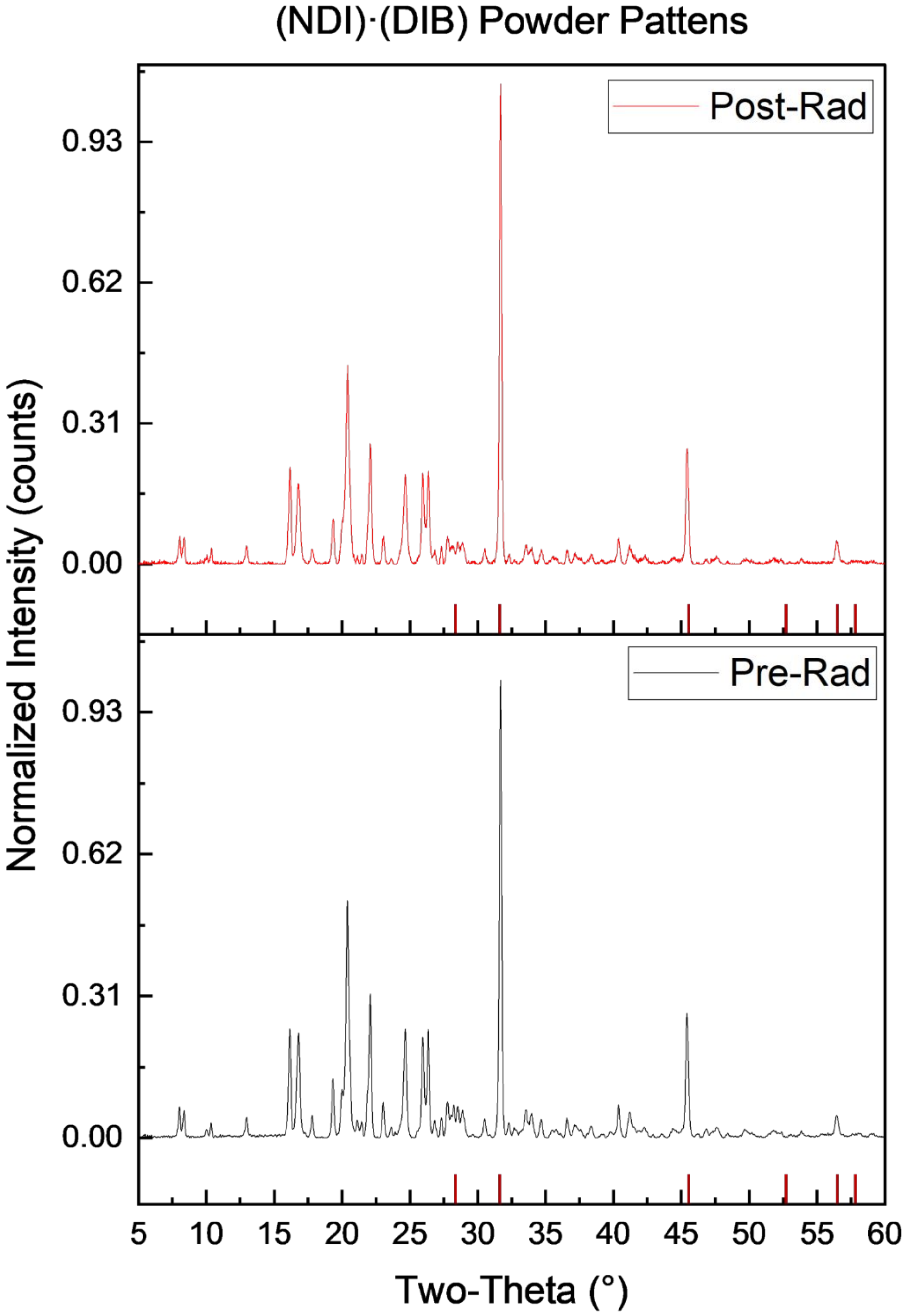

Figure S9. Normalized powder patterns pre- (black) and post-irradiation (red) for (**NDI**)·(**DIB**). NaCl standard powder pattern peaks are labeled with red tick marks.

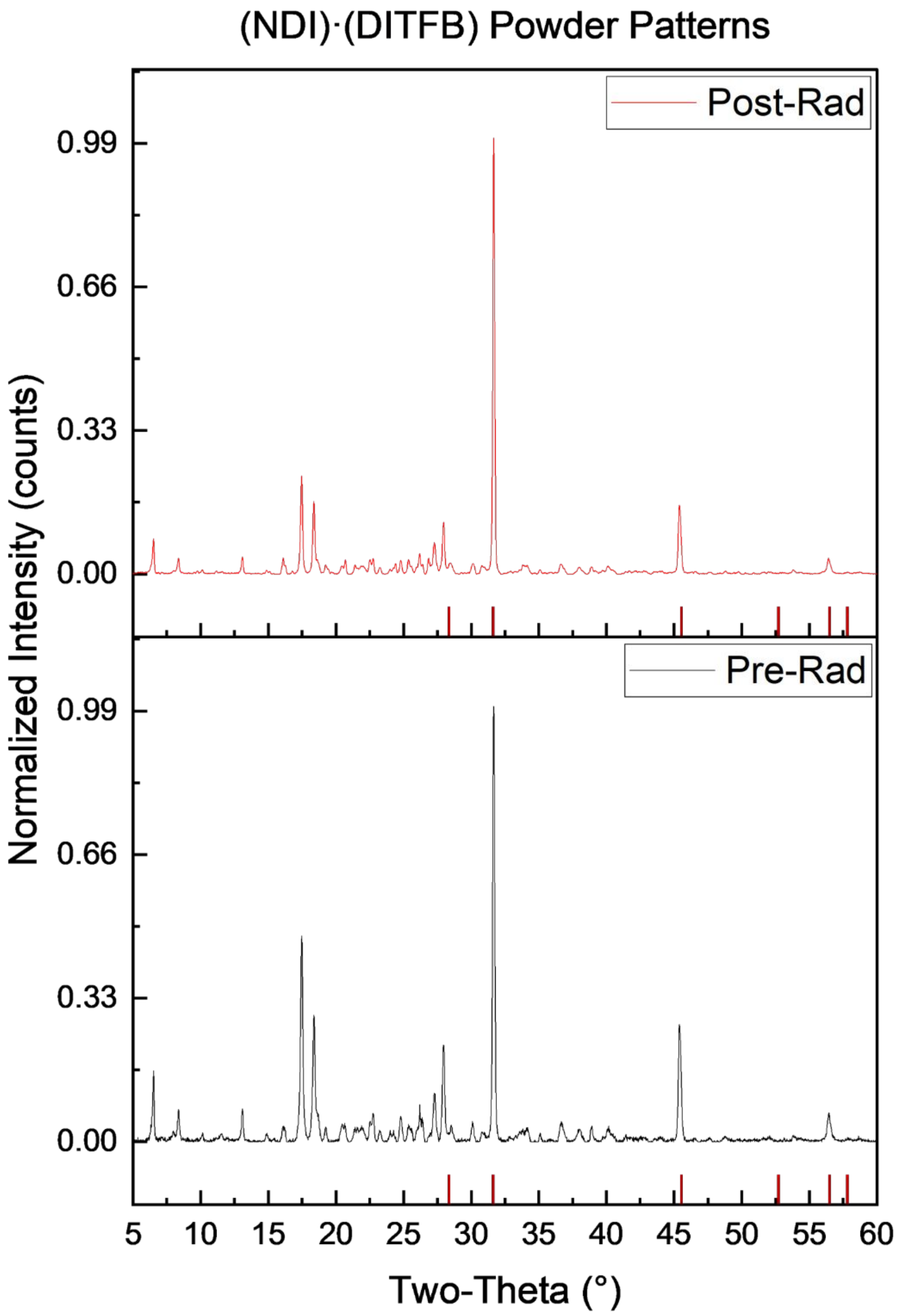

Figure S10. Normalized powder patterns pre- (black) and post-irradiation (red) for (**NDI**)·(**DITFB**). NaCl standard powder pattern peaks are labeled with red tick marks.

Pre- and post-irradiation diffractograms raw files

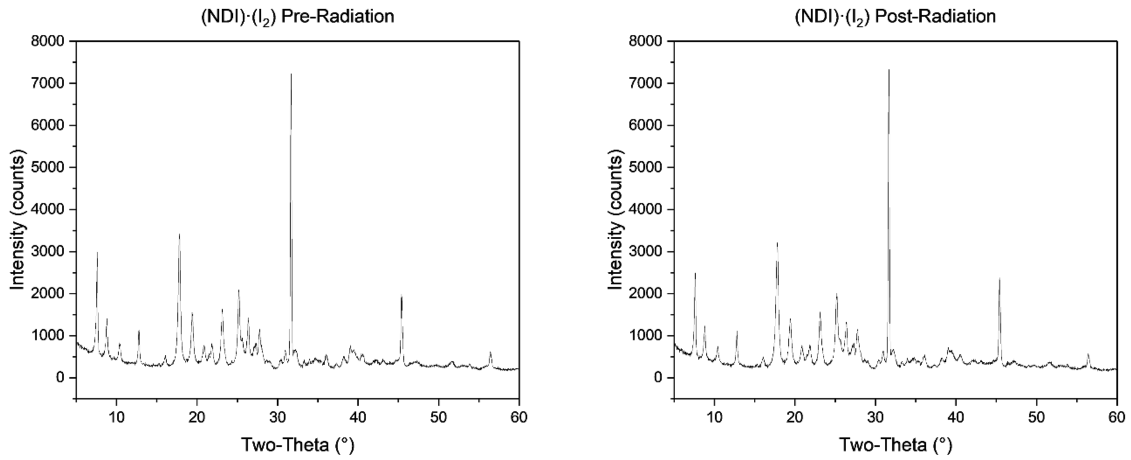

Figure S11. Raw powder patterns pre- (left) and post-irradiation (right) for (**NDI**)·(**I$_2$**).

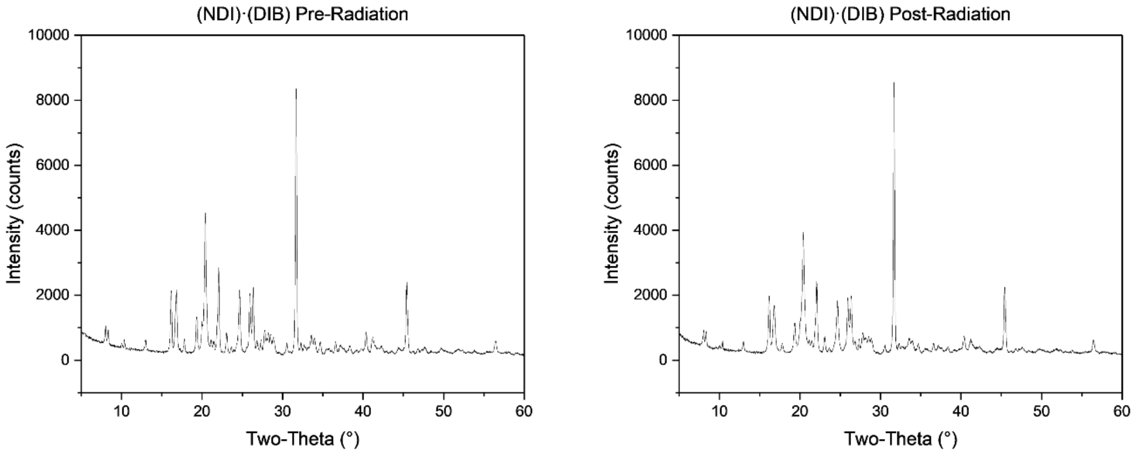

Figure S12. Raw powder patterns pre- (left) and post-irradiation (right) for (**NDI**)·(**DIB**).

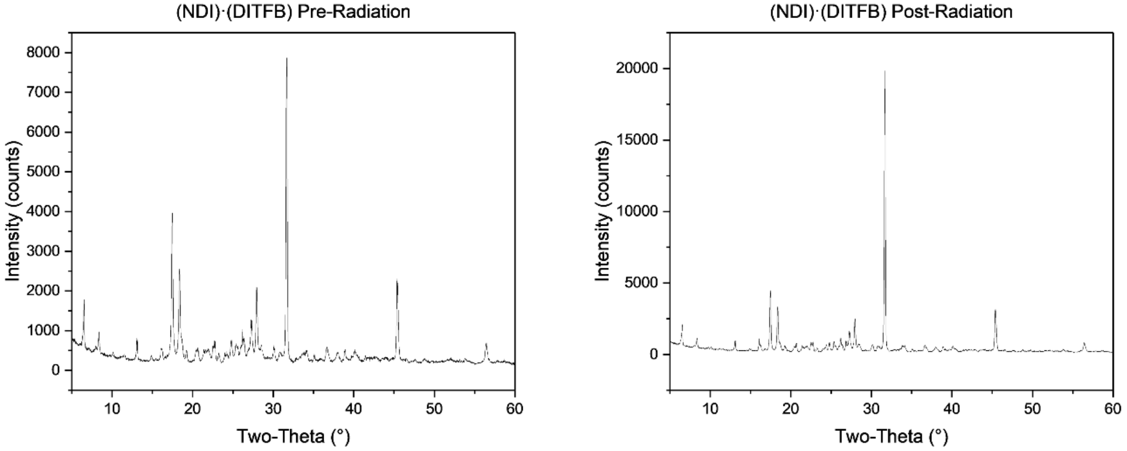

Figure S13. Raw powder patterns pre- (left) and post-irradiation (right) for (**NDI**)·(**DITFB**).

Calculated percent changes in crystallinity

Table 1. Intensity changes in powder patterns for (**NDI**)·(**I_2**).

2-Theta (□)	hkl	Pre-radiation Intensity (counts)	Post-radiation Intensity (counts)	Pre-radiation Intensity / NaCl (counts)	Post-radiation Intensity / NaCl (counts)	Percent Change (%)
7.62	001	2349.2	1982.4	0.34600	0.28398	17.926
17.81	021	3050.9	2734.1	0.44936	0.39166	12.839
25.18	221	1688.8	1379.2	0.24874	0.19757	20.570

Table 2. Intensity changes in powder patterns for (**NDI**)·(**DIB**).

2-Theta (□)	hkl	Pre-radiation Intensity (counts)	Post-radiation Intensity (counts)	Pre-radiation Intensity / NaCl (counts)	Post-radiation Intensity / NaCl (counts)	Percent Change (%)
16.16	002	1932.7	1600.8	0.23913	0.193865	18.930
20.4	02-1	4194.4	3262	0.5189	0.39504	23.87
22.08	11-1	2539.6	2035.8	0.31422	0.246546	21.538

Table 3. Intensity changes in powder patterns for (**NDI**)·(**DITFB**).

2-Theta (□)	hkl	Pre-radiation Intensity (counts)	Post-radiation Intensity (counts)	Pre-radiation Intensity / NaCl (counts)	Post-radiation Intensity / NaCl (counts)	Percent Change (%)
6.51	001	1234.9	1502.7	0.16251	0.08100	50.156
17.47	021	3554.1	4183.3	0.46773	0.22550	51.788
18.36	200	2208.4	3081.4	0.29063	0.16610	42.847

hkl planes

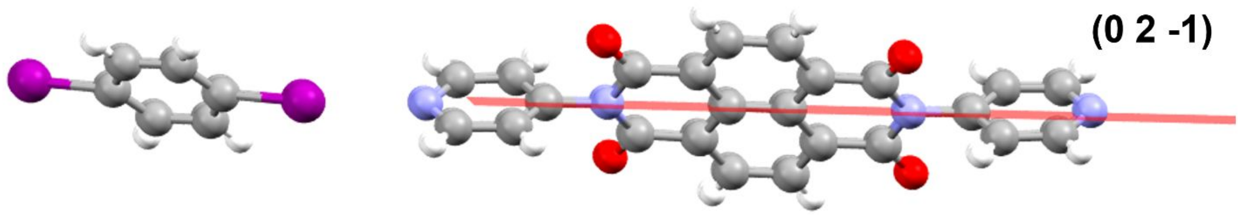

Figure S14. (0 2 -1) *hkl* plane of (**NDI**)·(**DIB**).

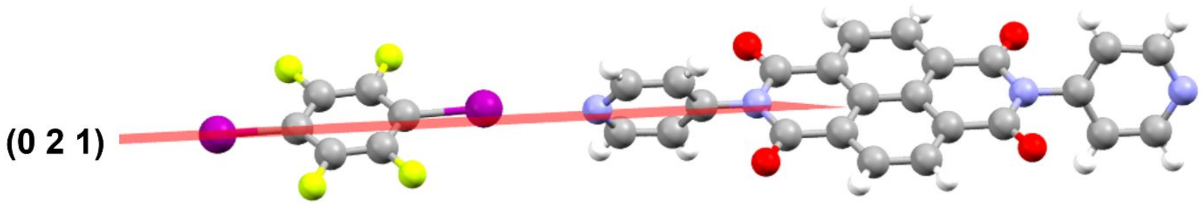

Figure S15. (0 2 1) *hkl* plane of (**NDI**)·(**DITFB**).
